# An End-to-End Trainable Multi-Column CNN for Scene Recognition in Extremely Changing Environment

**DOI:** 10.3390/s20061556

**Published:** 2020-03-11

**Authors:** Zhenyu Li, Aiguo Zhou, Yong Shen

**Affiliations:** 1 School of Mechanical Engineering, Tongji University, Shanghai 201804, China; zhenyu.li@tongji.edu.cn; 2 School of Automotive Studies, Tongji University, Shanghai 201804, China; shenyong111@139.com

**Keywords:** scene recognition, multi-column CNN, image retrieval, end-to-end trainable network

## Abstract

Scene recognition is an essential part in the vision-based robot navigation domain. The successful application of deep learning technology has triggered more extensive preliminary studies on scene recognition, which all use extracted features from networks that are trained for recognition tasks. In the paper, we interpret scene recognition as a region-based image retrieval problem and present a novel approach for scene recognition with an end-to-end trainable Multi-column convolutional neural network (MCNN) architecture. The proposed MCNN utilizes filters with receptive fields of different sizes to have Multi-level and Multi-layer image perception, and consists of three components: front-end, middle-end and back-end. The first seven layers VGG16 are taken as front-end for two-dimensional feature extraction, Inception-A is taken as the middle-end for deeper learning feature representation, and Large-Margin Softmax Loss (L-Softmax) is taken as the back-end for enhancing intra-class compactness and inter-class-separability. Extensive experiments have been conducted to evaluate the performance according to compare our proposed network to existing state-of-the-art methods. Experimental results on three popular datasets demonstrate the robustness and accuracy of our approach. To the best of our knowledge, the presented approach has not been applied for the scene recognition in literature.

## 1. Introduction

With the rapid development of machine learning and artificial intelligence, the application of visual robots has attracted wide attention [1,2]. In particular, visual robots are applied in the field of autonomous navigation, that is motivated by promising application in future autonomous driving. In order to enable robots to deal with problems autonomously in special environments, it is very important for visual robots have the ability to identify scenes they have visited. However, the robot will face many challenges in autonomous navigation [3]. One of the most difficult problems is how to achieve accurate scene recognition in unpredictable and complex environments. In most cases, illumination or viewpoint will change dramatically, which has significant impacts on results of scene recognition. In addition, different scenes with similar appearance will be a large challenge for robot to recognize.

**Figure 1:**
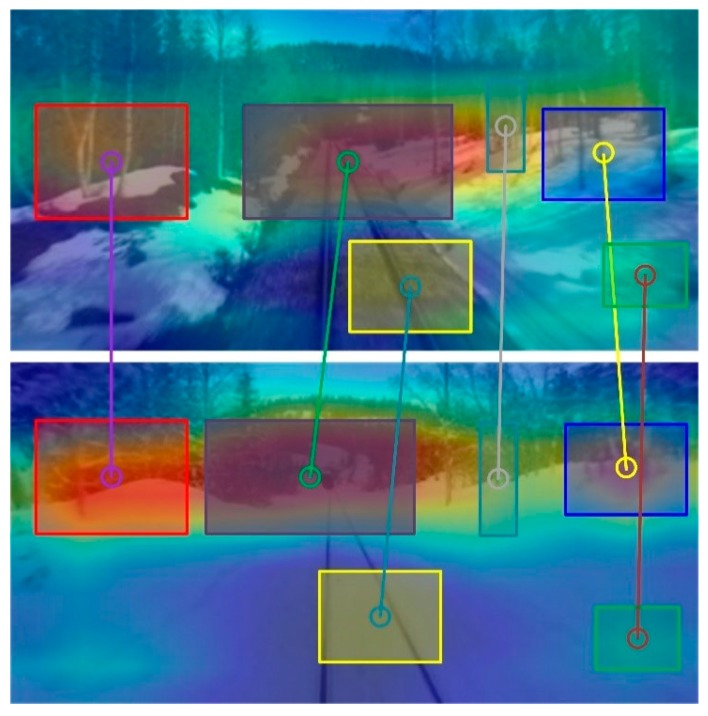
Robot recognizes the visited scene by matching local regions in a changing environment (e.g., Winter and Autumn). This method can be widely used to local region detectors, such as key points, segmentation method and object proposals. The rectangle of different colors represents the different position of interests.

In recent years, many algorithms have been applied to scene recognition [4,5,6,7], and one of the most popular are Convolutional Neural Networks (CNNs). CNNs are a deep learning method specially designed for image classification and image recognition based on multi-layer neural network. Due to the limitation of spatial structure and computational consumption, the traditional multi-layer neural network cannot meet the basic needs of robot navigation, however, the emergence of CNNs effectively solves these problems. The most frequently used networks are AlexNet [8], VGGNet [9] and GoogleNet [10] in the CNN family. Many researchers apply these networks to image classification, object detection and scene recognition. Compared with traditional neural networks, they absolutely improve the efficiency and performance of feature extraction and network training. In this paper, we proposed a novel approach for scene recognition with an end-to-end trainable multi-column CNN. Our proposed network structure is specifically designed for scene recognition capability of detecting visited scenes under extreme changes in indoor or outdoor, which combines the robustness against the appearance and viewpoint changes of CNN descriptors of local regions, as shown in Figure 1.

**Figure 2:**
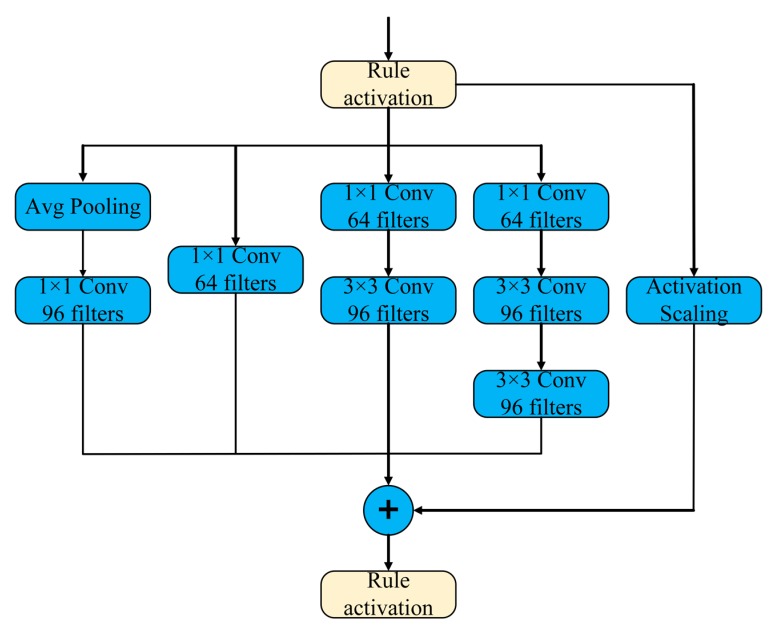
The architecture of Inception-A block from Inception-V4.

Compared with previous CNN-based methods, our proposed method consists of multi-column network and has a multi-level perception ability, which integrates the advantages of kinds of network and also takes full account of stability of feature extraction and feature representation. In addition, excluding the general networks using Softmax Loss to optimize the parameters, our proposed method takes L-Softmax Loss as back-end to optimize the parameters, which make extracted feature more discriminated, and hence significantly improving the performance on a variety of image retrieval and verification tasks. In summary, the novel contributions of our paper are as follows:

Firstly, an end-to-end Multi-column CNN is proposed, which takes VGG16 as its front-end and modified Inception-V4 (Inception-A) modules as its middle-end [11]. The architecture as shown in Figure 2. This network utilizes filter of different size to deal with the scale and viewpoint change in a complex environment, as well as has a strong ability of multi-level and multi-layer scene perception.

Secondly, we cast scene recognition as an image retrieval problem. In our view, the scene where the robot has visited are regarded as a series of datasets images, and the scene where need to be detected regarded as query image. Therefore, the key of scene recognition is how to find an image from datasets that is most similar to the query image.

Thirdly, in order to strengthen the discriminatory learning ability of network features, a novel L-Softmax Loss is used as back-end of the proposed network, which is able to not only adjust the desired margin but also avoid overfitting. According to [12], deeply learned features with L-Softmax Loss becomes more discriminating, which is helpful to distinguish the different scenes information.

The rest of this paper is organized as follows. We describe the related research on visual scene recognition with different kinds of feature representation in Section 2. Section 3 introduces the process of network training and image retrieval method. Experimental results are discussed in Section 4. Finally, we conclude the paper and propose the future work in Section 5.

## 2. Related Work

Scene recognition is a relevant and frequently studied problem in the robot application community [5,13]. It is the most important part of autonomous navigation of the robot. So far, many methods have been developed for scene recognition. In summary, the method of scene recognition can be categorized into three classes: Handcrafted method, which mainly used previous works. Sequence-based method, which use image sequence to retrieve image rather feature extraction. Lastly, CNN-based methods, which can automatically extract features without labelling them.

### 2.1. Handcrafted Feature Method

In the early stage, researchers mostly focused on the influence of image scale and rotation on scene recognition. In the process of robot motion, due to the influence of camera parameters and camera vibration, the extracted features will change greatly in scale. In order to cope with these problems, the approach of Scale-invariant feature transform (SIFT) is proposed [14]. The SIFT feature is just related to points of interest in the local appearance of the object, regardless of the size and rotation of the image, therefore, this approach to recognition can robustly identify scenes. However, SIFT algorithm relies heavily on the acceleration of hardware and the matching of special image processors. It is difficult for ordinary computer to real-time extract SIFT features. Speeded-Up Robust Features (SURF) drew on the idea of simplified approximation in SIFT, simplifying the Gauss two order differential template in DoH, that greatly improved the speed of feature extraction [15]. At the same time, ORB-SLAM approach [16] for scene recognition was proposed, which has been embedded a bag of words scene recognition module to perform relocation. This is a compromise approach, which takes full account of computational complexity and stability of feature expression. However, the above approaches use local descriptors to represent an image, which have difficulty in extracting key-points under lacking sufficient textures. Global descriptors can be a better alternative, so Histogram of oriented gradients (HOG) is widely used for scene recognition, which uses gradients to represent scene [17]. Although these methods of manual features perform well in complex environment, they still have many limitations. For example, handcraft feature has a poor scale invariance. However, the method of deep learning has a large advantage that the absolute scale can be learned from the large number of images, therefore, it can be predicted just by utilizing a single image without the need for scene based assumptions or geometric constraints.

### 2.2. CNN-Based Method

In recent years, the application of deep learning to image representation has attracted more and more attention. The appearance of CNN has played a ground-breaking role in the representation of image features. Compared with traditional handcrafted methods (e.g., SIFT, SURF and ORB-SLAM) and sequence-base method, CNN-based methods can automatically extract features and learn feature representation based on these features. It is clear that the CNN-based methods outperform the previous works. Based on CNN networks, researchers proposed many methods for scene recognition, such as graph-based CNN [18], light-weight CNN [19] and VLAD-based CNN [20]. The graph-based CNN is constructed by combining the features extracted from CNN and the temporal information of the images in a sequence, and the graph just includes nodes and edges, which greatly reduces computational consumption. Compared with CNN based on graph optimization, the use of light weight CNN in scene recognition is more concise and effective, by reducing layers and filters in the structure of CNN, which reduce the computational complexity greatly. In order to deal with the problem of large scale visual scene recognition, a kind of CNN structure that utilizes an end-to-end manner directly for the scene recognition task is proposed. The VLAD layer is integrated into CNN structure, which can be applied to very large-scale weakly labelled tasks. However, the above mentioned CNN models are all based on the original CNN structure, such as AlexNet and VGG, which will produce many parameters in the process of training. In the case of significant changes in the appearance of the scene, which poses a challenge to large-scale visual location recognition. In the literature [21], a new scene recognition approach is proposed, which combines an efficient synthesis of novel views with a compact indexable image representation. In the literature [22], a new scene recognition method based on end-to-end multimodal CNN is described. The context information is in the form of semantic segmentation, which using the information in the semantic representation to extract features from a series of RGB image. This control process enhances the learning of indicative scene content and enhances scene disambiguation by refocusing CNN’s reception domain. In the literature [23], A new method of indoor scene recognition is proposed, which exploits rich mid-level convolutional features to categorize indoor scenes. In addition, an image retrieval method based on depth space matching is proposed, in which image descriptors extracted from convolutional neural network activation based on global pooling [24]. In the last few years, a new modular network structure has been proposed, that is, the Google network series (include Inception-V1, Inception-V2 [25], Inception-v3 [26] and Inception-V4), which is a structure of Network in Network, and the number of its parameters is far less than VGG (about 5 million). In the structure of GoolgeNet, the original node is also a network. So far, the GoogleNets have been successfully applied to the field of the image classification. The previous works show that the prediction accuracy is improved while the parameters are greatly reduced. In our work, we proposed a novel visual scene recognition approach, which is greatly improved in recognition accuracy. The proposed approach uses an end-to-end multi-column network to improve the performance of feature extraction as well as maintain the stability of feature representation.

## 3. Proposed Approach

In this section, we introduce the structure of the network, and describe how the network is specially trained for scene recognition and learning feature representation.

### 3.1. The Multi-Column Network Structure

**Figure 3:**
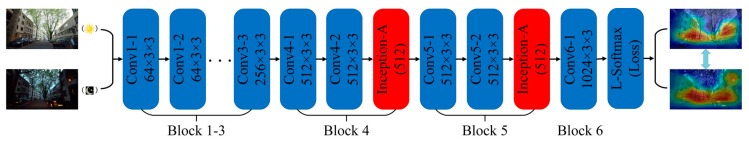
The multi-column network architecture. In the first 3 blocks (including 7 convolution layers), VGG16 pre-trained on ImageNet is used, and the parameters in the rectangle are represented as Conv − (block number) − (layer number) (filter number × filter size × filter size). In the next 3 blocks (also including 7 convolution layers), Inception-A modules are used, and the parameters in the rectangle are denoted as same with VGG16 modules.

The proposed network is a multi-column architecture, which is trainable end-to-end. The whole structure consists of three parts: front-end, middle-end and back-end. In Figure 3, the first three blocks make up the front-end of the network, blocks 4–6 make up the middle-end of the network and L-Softmax layer makes up the back-end of the network. In the convolutional process, the same size 3 × 3 convolutional kernel is used in front-end. Due to the better ability of perception in the middle layer, size 1 × 1 and 3 × 3 convolutional kernels are used in the middle-end, which is helpful for multi-level and multi-layer perception in large scale scenes. However, the structure in Figure 3 above cannot express the real network, to demonstrate the process more briefly, the process of pooling is not demonstrated in the figure, but it is real and exists. In this paper, we utilize maxpooling to aggregate all descriptors falling into that region to create a feature vector. Considering the complexity of network computing, it is beneficial to adopt the pre-trained model. In [27,28], a pre-trained CNN is used to extract features for scene recognition, which shows the perfect performance and efficiency. In our work, we adopt pre-trained VGG16 that is trained on the ImageNet dataset, and take it as the front-end of the whole structure, which is able to produce a compact image representation. Specifically, Inception-A modules are embedded into middle-end, and then trained in a distributed way, which is able to divide each copy into a model with multiple subnetworks to meet the memory requirement. At the back-end of the network, L-Softmax layer is used to effectively guide network learning, which is able to make the distance within the same class smaller and the distance outside the different class larger.

### 3.2. Train the Network by an Embedded L-Softmax Layer

**Figure 4:**
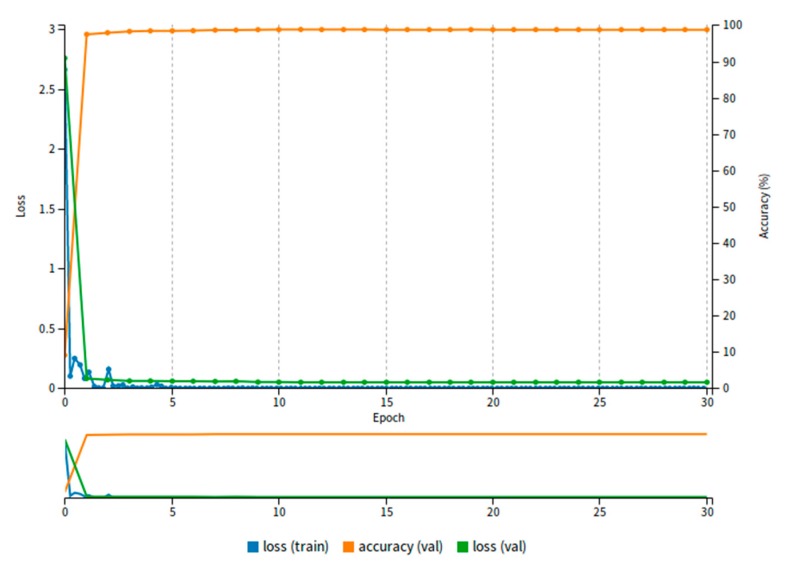
The changes in the value of L-Softmax loss as the increasing of training epoch on the KITTI dataset.

**Figure 5:**
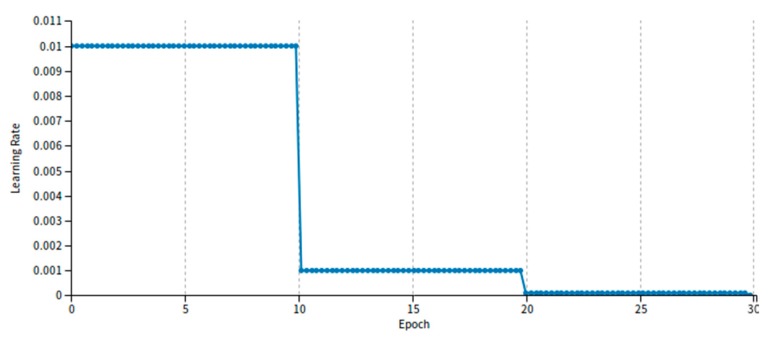
The changes in the value of learning rate with increasing training epoch on the KITTI dataset.

The Softmax Loss function is often used in convolutional neural network. It plays an essential role in image classification, object detection and scene recognition. It is simple and practical, but it cannot clearly guide the network learning to distinguish the features with high discrimination. However, the literature [29] represented Large-Margin Softmax (L-Softmax) that can effectively guide network learning and be able to make the intra-class distance smaller quickly. In addition, L-Softmax can not only adjust different margins, but also prevent overfitting, which is helpful to network training efficiency. Its forward and backward feedback can be calculated by using the stochastic gradient descent method. It can be seen from the Figure 4, with the increasing of the epoch, the value of loss (training and validation) exhibits a rapid reduction, and the opposite the value of accuracy presents a rapid rise within the range of training epoch. However, after this range is exceeded, the value of accuracy and loss remain almost unchanged, which demonstrates the excellent performance using L-Softmax loss function. We also show the changes in the value of learning rate with increasing training epoch on the KITTI dataset as shown in the Figure 5.

Before introducing L-Softmax, it is vital to review the traditional Softmax function. When defining the *i*th input feature $x_{i}$ and its label $y_{i}$, Softmax loss is recorded as:

(1) $$L=\frac{1}{N}\sum_{k=1}L_{i}=\frac{1}{N}\sum_{k=1}-\log\left(\frac{e^{f_{y_{i}}}}{\sum_{j}e^{f_{j}}}\right)$$

where $f_{j}$ denotes the *j*th element of the feature vector representing the output of the last convolution layer, and N is the number of training samples. We also define the activation function as W, therefore, we obtain the formula $f=W_{y_{i}}^{T}x_{i}$, and the final loss function can be expressed as:

(2) $$L=-\log\left(\frac{e^{\left\|W_{y_{i}}\right\|\left\|x_{i}\right\|\cos\left(\theta_{y_{i}}\right)}}{\sum_{j}e^{\left\|W_{j}\right\|\left\|x_{i}\right\|\cos\left(\theta_{j}\right)}}\right),0<\theta_{j}<\frac{m}{\pi }$$

The initial motivation of the Softmax is to obtain the inequality $\left\|W_{i}^{T}\right\|\left\|x_{i}\right\|\cos\left(\theta_{i}\right)>\left\|W_{i+1}^{T}\right\|\left\|x_{i+1}\right\|\cos\left(\theta_{i+1}\right)$, However, the key of large-margin Softmax Loss is that adding a positive integer variable m to generate a decision margin, which is able to more strictly constrain the above inequality, namely:

(3) $$\left\{ \begin{array}{l} a=\left\|W_{i}^{T}\right\|x_{i}\cos(\theta_{i})\\ b=\left\|W_{i}^{T}\right\|x_{i}\cos(m\theta_{i})\\ c=\left\|W_{i+1}^{T}\right\|x_{i+1}\cos(\theta_{i+1}) \end{array}\right.$$

If $W_{i}$ and $W_{j}$ can meet the inequality a>b, then a>c must be meted. Such constraints impose higher requirements on the learning process of $W_{i}$ and $W_{j}$, which making class 1 and class 2 have wider classification decision boundaries. Then, L-Softmax Loss function can be shown as Formulas (4) and (5):

(4) $$L_{i}=-\log\left(\frac{e^{\left\|W_{y_{i}}\right\|\left\|x_{i}\right\|\varphi \left(\theta_{y_{i}}\right)}}{\left\|W_{y_{i}}\right\|\left\|x_{i}\right\|\varphi \left(\theta_{y_{i}}\right)+\sum_{j\neq y_{i}}e^{\left\|W_{j}\right\|\left\|x_{i}\right\|\cos\left(\theta_{j}\right)}}\right),0<\theta_{j}<\frac{\pi }{m}$$

(5) $$\varphi \left(\theta \right)=\left\{ \begin{array}{l} \cos\left(m\theta \right),0<\theta <\frac{\pi }{m}\\ \vartheta \left(\theta \right),\frac{\pi }{m}<\theta <\pi \end{array}\right.$$

When m gradually becomes bigger, this causes the boundary of classification to also be bigger and the learning difficulty is higher. Most importantly, φ(θ) must be a Monotone decreasing function and meet equation $\vartheta \left(\theta \right)=\cos\left(\frac{m}{\pi }\right)$, which makes sure that φ(θ) is a continuous function.

### 3.3. Image Retrieval

**Figure 6:**
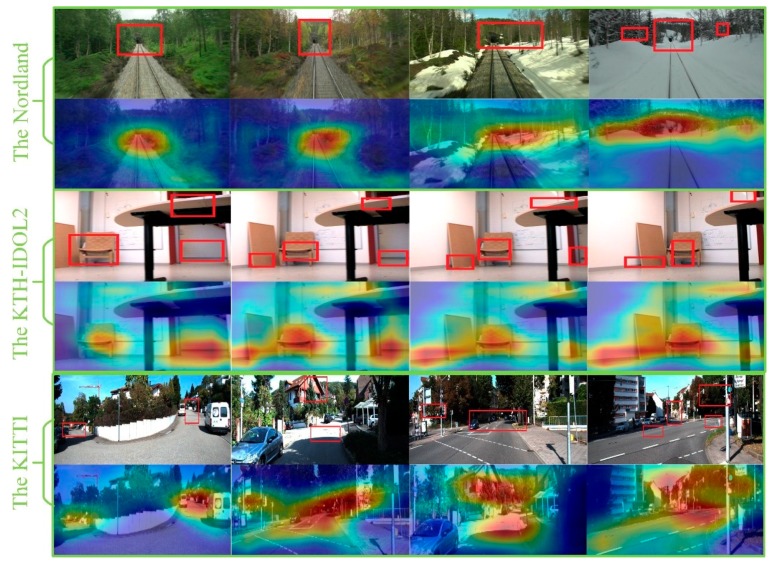
Recognized salient regions with our proposed method. Image samples come from three different datasets. The red rectangular represents salient region and highlighted regions represent salient regions recognized.

In the paper, extracted features are used to provide the spatial image support for scene recognition, which is the local descriptors in the perceptual field of the image. Given an image, it has been known that local pixels are closely related while the remote pixels are weakly correlated. Therefore, it is not necessary for each neuron to perceive the whole image. Instead, it only needs to perceive these regions of local interests, and then the local information can be integrated at a higher level for obtaining the global information. The main function of the feature detector is to provide spatial image support for the subsequent description steps. To tackle the problem of changes in appearance and viewpoint because of the scale and illumination changes, the proposed multi-column network is trained for representing the interest regions. The first step in visual scene recognition is that an image is directly fed into the multi-column network, and then robust features are able to be extracted. The second step, all extracted descriptors in an image are fed into the back-end of the network, and then salient descriptors begin to aggregate together, which is able to represent an interested region in an image, as shown at the highlight regions in Figure 6. The last step, to retrieve an image, here we define xi as one of the descriptors in the image A and yi as one of the descriptors in the image B. The match between image A and image B is performed by matching all region vectors $P^{A}=\left(x_{1},x_{2},...,x_{n}\right)$ and vectors $P^{B}=\left(y_{1},y_{2},...,y_{n}\right)$. In our work, we utilize cosine similarity for image retrieval, the similarity of regions between region i from image A and region j from image B can be calculated according to the Formula (6):

(6) $$S=\frac{\sum_{i=1,j=1}^{M}\left(x_{i}\times y_{j}\right)}{\sqrt{\sum_{i=1}^{M}{\left(x_{i}\right)}^{2}}}=\frac{P_{i}^{A^{T}}P_{j}^{B}}{\left\|P_{i}^{A}\right\|\left\|P_{j}^{B}\right\|}$$

where i=1,2,...,M, and i,j denote ith and jth salient regions in image A and image B, respectively. According to the Formula (6), the high similarity regions in the two images can be matched. Then, the weight in the process of extracting each local feature can be expressed as:

(7) $$W^{\prime }=\mathrm{lg}\left(\frac{k}{n_{c}}\right),c=1,2,...,N$$

where k is the total number of training images, and $n_{c}$ is the number of images containing the region c. However, in order to determine the similarity between two images A and image B, all similarity of salient regions in both images should be calculated. In our work, we adopt Cross-checking principle to complete the overall similarity between two images A and image B:

(8) $$Q_{A,B}=\frac{1}{M}\sum_{i,j}S_{i,j}\times W_{i}\times W_{j}$$

where $W_{i}{}^{\prime }$ and $W_{j}{}^{\prime }$ is the weight of feature vectors $P^{A}$ and $P^{B}$, respectively. Then, in order to search the best matched reference image A corresponding to image B from a dataset, all referenced images in the dataset are traversed, and the image with the highest similarity score is selected that realizes scene recognition. The highest similarity score can be calculated by the Formula (9):

(9) $$\Omega \left(B\right)=\arg\underset{A}{\max}Q_{A,B}$$

L-Softmax Loss has a clear geometric explanation and that can adjust the difficulty of training by setting the value of m. It is able to effectively prevent over-fitting, and effectively reduce the intra-class distance and increase the inter-class distance.

## 4. Experimental Results and Analysis

In this section we describe the experimental setup and results evaluation. In the experiment, we use a computer equipped with an i9-processor and 1080Ti graphics (11G) card to train our proposed network. Meanwhile, in order to demonstrate the superiority of our proposed method, other experiments are also completed on the same hardware platform, and then the performance of our proposed method compared with state-of-art. In order to give quantitative and qualitative results, we compare our proposed method with others on three standard scene recognition benchmarks.

### 4.1. Performance Measurements

The proposed method was evaluated against other state-of-the-art algorithms of scene recognition. The performance evaluation method we adopt is Precision–Recall curves. In our experiment, we compare our proposed method with hand-crafted feature method and CNN-based feature method, such as SeqSLAM, VGG and NetVLAD. We also exhibit the visual detection results in three datasets, as shown in Figure 6. We assume that TP denotes true positive, TN denotes true negative, FP denotes false positive and FN denotes false negative, then the precision (A) and recall (B) can be calculated:

(10) $$P=\frac{TP}{TP+FP}$$

(11) $$R=\frac{TP}{TP+FN}$$

### 4.2. Dataset Used in the Experiment

In the experiment, three popular datasets are used, we divide them into three groups according to changes in appearance and viewpoint. The Nordland Dataset exhibits severe appearance change while virtually no variation in viewpoint. On the contrary, the KTH-IDOL2 dataset exhibit severe viewpoints change, but no variation appearance change. The last dataset is a compromise choose, we choose KITTI dataset as an independent group mainly because it does not show much change in appearance or viewpoints. The specific character of the three datasets are shown in Table 1.

#### 4.2.1. The Nordland Dataset

The Nordland dataset is collected along railway lines from the perspective of the front cart, consists of about 10 h of video in four different seasons. The Nordland dataset exhibits severe appearance change that occur when the seasonal change from spring to winter, and it is a perfect experimentation dataset since it’s almost no change in viewpoint.

#### 4.2.2. The KTH-IDOL2 Dataset

The KTH-IDOL2 dataset is collected in indoor environments by laser scanning. It consists of 24 image sequences. All image sequences are continuous acquainted at the rate of 5 fps under different illumination. Each image sequence exhibits severe viewpoint, in order to make sure that the experiment is carried out in an environment where there is no change in the appearance but only in the viewpoint, we only select one of the sequences to carry out the experiment.

#### 4.2.3. The KITTI Dataset

The KITTI dataset is the largest computer vision algorithm evaluation dataset in the world under the circumstance of automatic driving, it totally consists of 22 stereo sequences, saved in a loss less png format. The first 11 sequences (00–10) with ground truth trajectories are used for training, and the next 11 sequences (11–21) without ground truth are used for evaluating. In our experiment, the image sequence (00) is chose for training, and the image sequence (10) is chose for test.

### 4.3. Scene Recognition with Appearance Change

**Figure 7:**
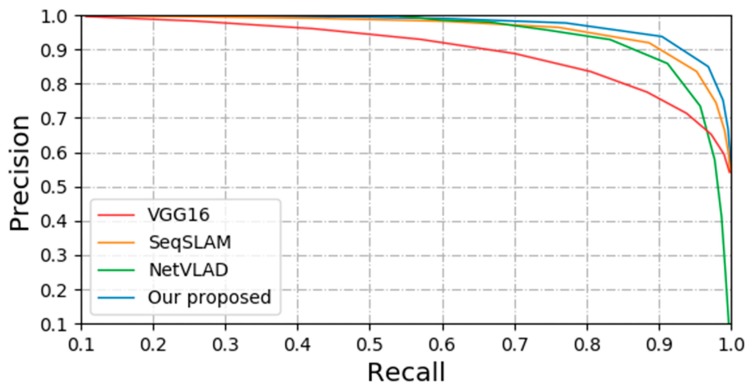
The performance comparison between our proposed method and other three methods in the Nordland dataset.

Figure 7 shows benefits of the presented approach using multi-column CNN compared to the best existing advanced matching approach in the Nordland dataset. The blue curve shows matching based on image descriptor of NetVLAD as described in [30]. This approach fails in the presence of the appearance changes greatly. The reason may be that its retrieval effect on a small database is better, but when the size of database becomes large, the retrieval effect using this retrieval algorithm is very unstable, and there may be a significant decline. The red curve shows matching based on image descriptor of VGG16. This approach exhibits stable performance in case of extreme changes in appearance. However, comparing to our proposed approach, the performance is not good. The main reason is that the network structure of classical VGG16 is relatively simple, which has the same size of filters so that only has a relatively simple receptive field, and the network we present is a multi-column structure that has different receptive field and corresponding to visual scene of different scales, as descripted in [29]. The orange curve shows matching based on method of SeqSLAM as described in [31]. This approach exhibits a better performance, which is almost equivalent to methods of CNNs. The main reason is that this method discards feature-based image matching for visual localization, but adopts a sequence-based approach. Under the conditions of extreme environmental changes, the authors of the proposed method verified their good performance.

### 4.4. Scene Recognition with Viewpoint Change

**Figure 8:**
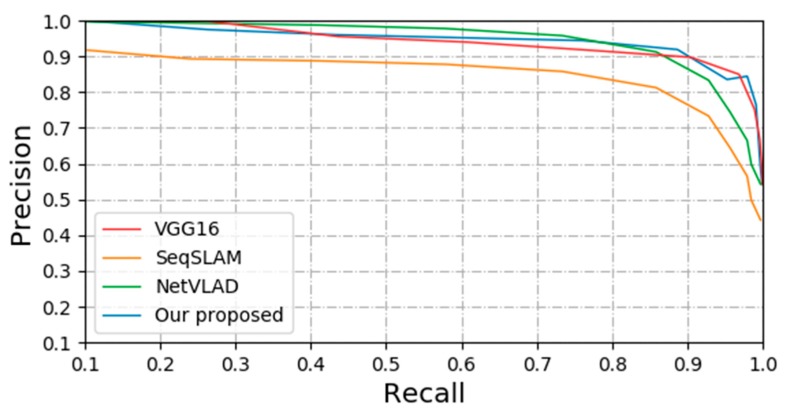
The performance comparison between our proposed method and the other three methods in the KTH-IDOL2 dataset.

Figure 8 shows the performance comparison between our proposed method and the other three methods in the extreme viewpoint changes. As can be seen from the four curves, the method based on SeqSLAM presents worse than the other three methods. The reason is that it is a sequence-based method for image matching, which has no feature self-learning ability and no receptive field to perceive each pixel in the image, and thus performs poorly in an environment where the viewpoint changes strongly. For other three methods, they are all CNN-based method for image matching, which have the ability that can automatically extract features and learn feature description based on these features. So these three methods perform better under viewpoint changes.

### 4.5. Scene Recognition with No Appearance and Viewpoint Change

**Figure 9:**
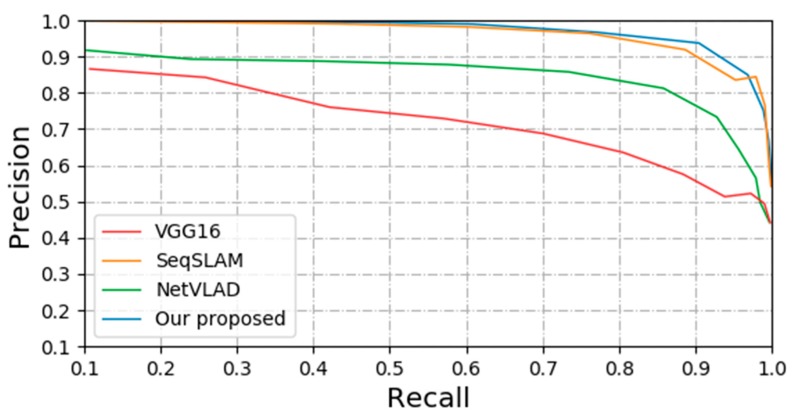
The performance comparison between our proposed method and the other three methods in the KITTI dataset.

Furtherly, in order to show the performance of the four methods, we select the KITTI dataset with no significant change in viewpoint and appearance for image matching experiment verification, as shown in Figure 9. It can be seen that the methods based on SeqSLAM and our proposed perform better. The main reason is that in the environment where the appearance and the viewpoint do not change greatly, the variation of feature scale presented in the captured image is not particularly serious, which makes the method based on the learning feature not necessarily better than the sequence-based method. The main reason for the different in performance between the three CNN-based methods may be that the three different methods have different structure of back-end. The method of NetVLAD and our proposed all use end-to-end training technology to learn the training parameters, which has great advantages in image classification and image matching.

### 4.6. Robustness Analysis

**Figure 10:**
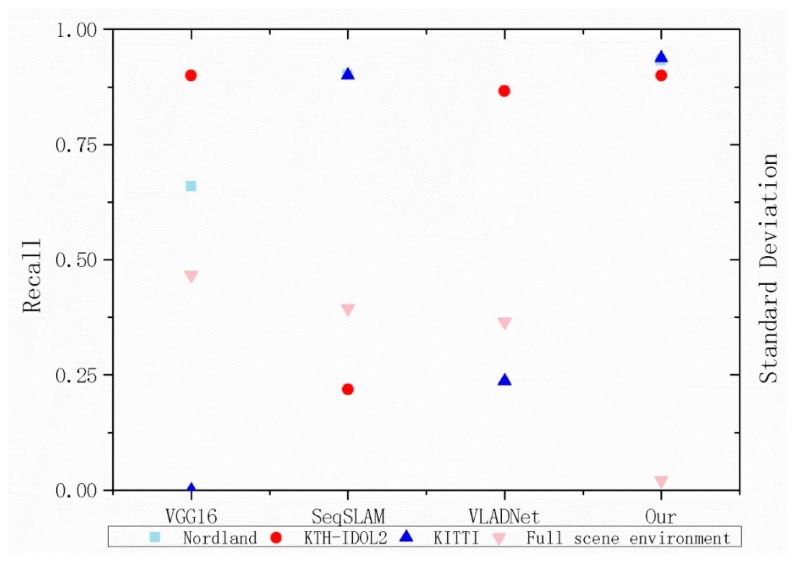
Recall and standard Deviation at 90% of precision of proposed method and the other three methods. Specifically, 0 recall at 90% of precision represents that the precision of the method has been below 90%.

In order to show the robustness of the four methods in different scenes, we compare the recall and standard deviation at 90% of precision of four methods. A larger standard deviation could have a better robustness. The standard deviation can be calculated as follows:

(12) $$H=\sqrt{\frac{\sum_{i=1}^{n}\left(X_{i}-M\right)}{n-1}}$$

where X is the recall rate. M is the average recall rate of four methods. It can be seen from Figure 10, compared with the other three methods, that the distribution of recall rate of our method is relatively centralized, which corresponds to the lowest value of standard deviation (0.02043). The discrete level of the VLADNet (0.36431) and SeqSLAM (0.39404) tends to be the same. The most serious discretization is VGG16 (0.4659). The main reason is that its performance on the KITTI dataset is poor. Compared with other datasets, The KITTI dataset has little changes in appearance or viewpoint, in fact, VGG16 has no advantage over other methods in this dataset. To sum up, our method has better robustness in a more complex environment, as well as has relatively stable performance in full scene environment.

### 4.7. Ablation Study

**Figure 11:**
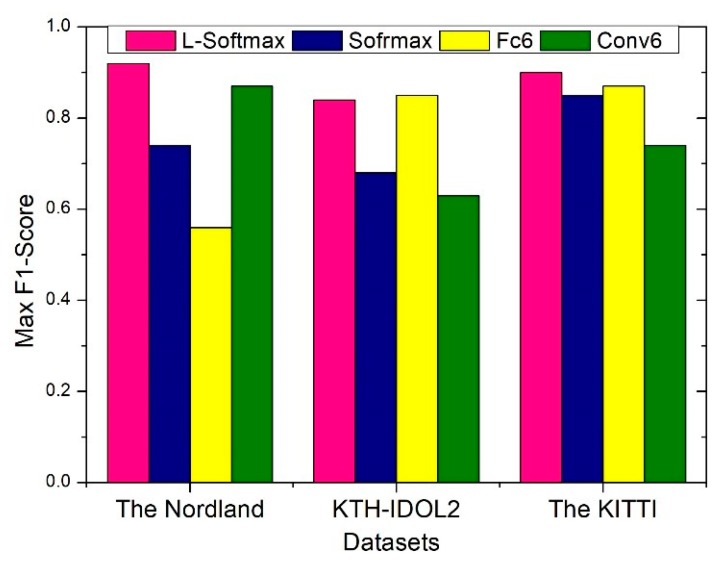
Different back-end components show different performance. Four kinds of structures have the same front-end and middle-end, in which VGG modules and Inception-A modules are included.

**Figure 12:**
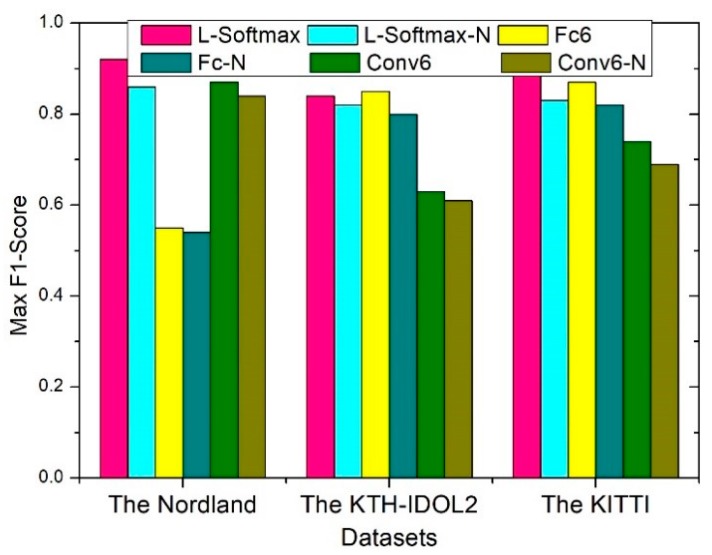
Different middle-end components show different performance. Six kinds of structures are sorted into three groups, in which each group has the same structure except middle-end. In each group, one has Inception-A modules and another one has no Inception-A module. For example, L-Softmax has Inception-A modules, and L-Softmax-N does not have Inception-A modules.

In order to verify the effect of each component of the proposed network on recognition performance, an extended ablation study is conducted in this section. This is similar to the control variable method in Mathematics. Firstly, while the front-end and middle-end of the proposed network remain structurally unchanged, the effect of different back-end structures on the entire network is demonstrated by comparing Max F1-Score. In Figure 11, four kinds of structure of back-end are showed, which all consist of the first five VGG modules and embedded Inception-A modules. The difference between them is that Fc6 adds three fully connected layers after the modules of the network and Conv6 just utilize six modules of the network. In addition, the former consists of an original loss function, which includes loss term and regularization term. The latter directly extracts feature vectors from the module that used to similarity calculate. As can be seen, by making learning harder, L-Softmax loss forces the model learn the distance between the classes become larger, and the distance within the class become smaller, which effectively improves the performance. Regardless of the scenario, the back-end that consists of L-Softmax perform better than others. Moreover, we compare the performance between the two structures with Inception-A modules and without Inception-A modules. We adopt the multi-column architecture with different filter size to deal with the scale and perspective change in complex environment, which is able to improve the detection precision, as shown in Figure 12. We can see that having the Inception-A modules performs better than having no Inception-A modules in every group.

## 5. Conclusions

In this paper, we proposed a novel approach for scene recognition with an end-to-end trainable multi-column CNN network. The multi-column CNN consists of some VGG16 layers, Inception-A modules and L-Softmax layer, which has a strong multi-level and multi-layer perception ability. Instead of the whole perceptual image, the proposed method is based on the detection of highly salient regions for scene recognition. We validate the proposed method on three representative datasets. The results show our proposed method is capable of successfully retrieving images. We also compared the performance between the proposed method and three other state-of-the-art methods, which includes CNN-based methods and hand-crafted method. The experimental results under the condition of obvious appearance change show that the proposed method is comparable to the state-of-the-art, and under the condition of sever viewpoint change also demonstrates a better performance, and under the condition of minor viewpoint change and no appearance changes shows better performance by using the proposed method. In addition, an extra ablation study is used, which verifies the role of various elements of our multi-column network. All experiments demonstrate that the proposed method is able to deal with the problems of scene recognition under appearance and viewpoint extreme change.

## Figures and Tables

**Table 1:** The dataset used in our experiment and show the change in appearance and viewpoint.

Dataset	Environment	Appearance	Viewpoint
The Nordland dataset	train journey	severe	minor
The KTH-IDOL2 dataset	indoor	minor	sever
The KITTI dataset	outdoor	none	minor
